# Disparities in health services and outcomes by National Health Insurance membership type for ischemic heart disease and stroke in Indonesia: analysis of claims, 2017–2022

**DOI:** 10.1186/s41256-025-00432-y

**Published:** 2025-08-01

**Authors:** Ede Surya Darmawan, Syarif R. Hasibuan, Vetty Yulianty Permanasari, Dian Kusuma

**Affiliations:** 1 Department of Health Administration and Policy, Faculty of Public Health, Universitas Indonesia, Depok, Indonesia; 2 Center for Health Administration and Policy Studies, Faculty of Public Health, Universitas Indonesia, Depok, Indonesia; 3 Faculty of Medicine, Universitas Pembangunan Nasional Veteran, Jakarta, Indonesia; 4 Department of Public Health and Epidemiology, College of Medicine and Health Sciences, Khalifa University of Science and Technology, Abu Dhabi, United Arab Emirates

**Keywords:** Health services, Hospital stays, Ischemic heart disease, Mortality, Socioeconomic status, Stroke

## Abstract

**Background:**

Ischemic heart disease (IHD) contributed to around 8.9 million deaths and stroke accounting for about 6.2 million deaths each year. This study examines disparities in health services and outcomes for IHD and stroke among different membership types within the national health insurance.

**Methods:**

We analyzed over 30,000 inpatient claim data for IHD and stroke patients from 2017 to 2022 in Indonesia. The associations were assessed between National Health Insurance (*Badan Penyelenggara Jaminan Sosial*, BPJS) membership types and five dependent variables including treatment/diagnosis, severity, mortality, length of stay, and claim cost. Membership types included the poorest members subsidized by the national budget (*Penerima Bantuan Iuran Anggaran Pendapatan dan Belanja Negara*, PBI APBN); near poor, subsidized by local governments (*Penerima Bantuan Iuran Anggaran Pendapatan dan Belanja Daerah*, PBI APBD); informal non-workers (*Bukan Pekerja*, BP), informal workers (*Pekerja Bukan Penerima Upah*, PBPU), and formal workers (*Pekerja Penerima Upah*, PPU).

**Results:**

For treatment access, PBI APBN members with IHD had lower odds of receiving percutaneous coronary interventions (PCI) compared to other groups, though this difference was not statistically significant in the multivariate models. For stroke patients, access to head computed tomography (CT) scans—critical for diagnosing stroke type—was similar across all membership types. Length of stay varied by condition; PBI APBN members experienced longer hospital stays for IHD but shorter stays for stroke. However, claim costs were significantly higher for non-subsidized groups (BP, PBPU, PPU) compared to the PBI APBN group for both IHD and stroke patients. Regarding health outcomes, non-subsidized IHD patients (BP, PBPU, PPU) had significantly lower odds of severe cases with adjusted odds ratios (AORs) of 0.70, 0.76, and 0.66, respectively, and mortality (AORs of 0.61 and 0.64 for BP and PPU) compared to the subsidized PBI APBN group. For stroke patients, although severity levels were comparable across membership types, non-subsidized patients (BP, PBPU, and PPU) had significantly lower odds of mortality, with AORs of 0.66, 0.73, and 0.54, respectively.

**Conclusions:**

Non-subsidized members had lower severity and mortality for IHD and stroke but higher treatment costs, while the poorest (PBI APBN) faced longer stays and worse outcomes—highlighting persistent disparities in Indonesia’s national health insurance system. Addressing these inequities requires targeted policies to improve access, care efficiency, and quality for the poorest populations. Strengthening community-based lifestyle promotion and tobacco control can further reduce the burden of IHD and stroke and help close these gaps over time.

**Supplementary Information:**

The online version contains supplementary material available at 10.1186/s41256-025-00432-y.

## Background

Cardiovascular diseases (CVDs) remain the leading cause of mortality globally, with a substantial burden on healthcare systems and economies. According to the World Health Organization (WHO), CVDs accounted for approximately 17.9 million deaths in 2019, representing 32% of all global deaths [1]. Ischemic heart disease (IHD) and stroke are the predominant contributors to these fatalities, with the former causing around 8.9 million deaths and the latter accounting for about 6.2 million deaths each year [1]. The latest Global Burden of Disease (GBD) Study showed that IHD and stroke, together with Coronavirus Disease 2019 (COVID-19), were the top three leading causes of deaths in 2021 [2]. These conditions are particularly prevalent in low- and middle-income countries (LMICs), where over 75% of CVD deaths occur [1].

In Indonesia, the burden of CVDs is particularly severe, even during and after the pandemic. According to the GBD study, stroke and IHD were the top two leading causes of deaths in 2021 [2]. The GBD study reported that in 2019, IHD caused approximately 245,000 deaths, while stroke accounted for around 331,000 deaths [3]. The latest Indonesia Health Survey (SKI) reported the prevalence of IHD and stroke at 8.5 and 8.3 per 1,000 population, respectively, in 2023 [4]. This high prevalence of chronic conditions is reflected in the claims made through the national health insurance system. In 2023, claims for IHD reached 20 million cases, costing IDR 17.6 trillion (around USD 1.1 billion), a substantial 50% increase from the IDR 12.1 trillion (USD 777 million) spent in 2022. Stroke also had a significant financial impact, with 3.46 million claims in 2023, amounting to IDR 5.2 trillion (approximately USD 334 million) [5].

The national health insurance program, *Jaminan Kesehatan Nasional* (JKN), administered by BPJS Kesehatan (*Badan Penyelenggara Jaminan Sosial*, BPJS), was launched in 2014 to achieve Universal Health Coverage (UHC) [6]. This program aims to provide essential health services to all Indonesians without financial hardship. BPJS serves as the payer and manages hospital claims within Indonesia's national health insurance program. BPJS membership is categorized into several types based on eligibility and funding sources: PBI APBN for subsidized members funded by the national budget targeting the poorest groups; PBI APBD for those subsidized by local government budget targeting the near poor; Informal non-workers (*Bukan Pekerja*, BP) for employers, investors, pensioners, and veterans informal; *Pekerja Bukan Penerima Upah* (PBPU) for informal workers who contribute directly; and *Pekerja Penerima Upah* (PPU) for formal workers with shared contributions between employees and employers [6]. This stratification is designed to accommodate various socioeconomic groups, thereby expanding coverage.

Despite these efforts towards UHC, significant socioeconomic and regional disparities in health services and outcomes persist, particularly among CVD patients. Globally, individuals from lower-income backgrounds often struggle to access specialized treatments like percutaneous coronary interventions (PCI) or advanced imaging for stroke [7]. In Indonesia, these disparities are compounded by geographic factors; urban populations generally have better access to healthcare facilities compared to rural residents [8–10]. Regions such as Java and Bali have more tertiary hospitals and specialist centers compared to less-developed areas like Papua and Maluku [11].

The existing literature on disparities in health services and outcomes for IHD and stroke among hospitalized patients, particularly those using health insurance claim data, faces several limitations. First, much of the research comes from high-income countries, such as South Korea and the United States, likely due to the availability of extensive claim data in these settings [12–14]. This geographic focus limits the generalizability of findings to LMICs, where healthcare systems and patient populations differ significantly. Second, many studies on CVD disparities in LMICs rely on alternative data sources, such as national health surveys, GBD data, and BioBank data [11, 15, 16]. While valuable, these data sources often have limitations, including recall bias related to self-reported hospitalizations and reliance on modeled estimations rather than actual utilization claim data. Finally, existing research that does use health insurance claim data frequently focuses on broader healthcare utilization and expenditures or on other conditions, such as diabetes and HIV, rather than on specific CVD outcomes like IHD and stroke [6, 17–19]. These gaps highlight the need for more research using claim data to better understand disparities in CVD care and outcomes in diverse contexts such as Indonesia. Addressing this gap, this study aims to examine disparities in health services and outcomes for IHD and stroke across different membership types within Indonesia’s national health insurance system.

## Methods

### Study design

This study employed a cross-sectional observational design, analyzing claim data from patients diagnosed with IHD and stroke between January 1, 2017, and December 31, 2022. The study included all patients aged 16 years and older. IHD was defined using the general International Classification of Disease, 10th Revision (ICD-10) codes I20, I21, I22, I23, I24, and I25, while stroke was defined using ICD-10 codes I60, I61, I63, and I64. ICD-10 codes used were based on the general ICD-10 classification system adopted nationally by BPJS, which follows the WHO ICD-10 coding [20].

### Data extraction

We analyzed over 30,000 inpatient claim data for IHD and stroke patients from 2017 to 2022 in Indonesia. Each claim represents an inpatient episode and not necessarily a unique patient, as the same patient may appear in the dataset more than once. These data are publicly available and anonymized to ensure no individual identifiers. The sampling process used families as the sampling units instead of individual participants. To capture sufficient data on service utilization, over-sampling was performed for participants who accessed services, given that the proportion of service users was smaller compared to non-users. This non-proportional sampling approach ensured adequate representation of service utilization behaviors [17].

BPJS provided sample data covering participants and services from 2015 to 2022, including an additional sample of new participants who enrolled in 2022. To reflect the most recent conditions, the sample included both newly registered members in 2022 and their corresponding service data for that year. A more detailed description of the data collection and sampling methodology is available in the official BPJS guidelines. For the analyses, the final dataset included 14,658 claims for IHD and 16,289 claims for stroke between 2017 and 2022.

### Variables

The study analyzed five outcome variables: treatment/diagnosis, severity, mortality, length of stay, and treatment costs. Treatment/diagnosis (1 = treatment, 0 = otherwise), severity (1 = severe/moderate, 0 = otherwise), and mortality (1 = died, 0 = otherwise) were binary variables, while length of stay and treatment costs were continuous. Length of stay was measured in days, and treatment costs were recorded in Indonesian rupiah (IDR), transformed using a natural logarithmic scale for the analyses. Hospitalization costs in this study reflect standardized DRG-based payments (INA-CBGs) as recorded in the BPJS claim system. These episode-level bundled payments are fixed by disease group, severity, hospital class, and procedure. As DRG tariffs did not change during the 2017–2022 period, inflation adjustments were not applied [19].

For IHD, PCI was selected as the treatment variable due to its importance, high cost, and relatively high prevalence in the BPJS claim sample (2013 claims, or about 14% of all IHD claims during the study period). PCI is commonly used to improve blood flow in narrowed coronary arteries, particularly for relieving symptoms of angina. For stroke, head computed tomography (CT) scans were chosen as a proxy for advanced treatment due to their prevalence (7422 claims, or 46% of all stroke claims) and their critical role in diagnosing the type of stroke (ischemic or hemorrhagic) and guiding treatment strategies [21].

The primary independent variable was BPJS membership type, categorized into five official groups: (1) *Penerima Bantuan Iuran Anggaran Pendapatan dan Belanja Negara* (PBI APBN) for subsidized members funded by the national budget (or APBN), targeting the poorest groups; (2) *Penerima Bantuan Iuran Anggaran Pendapatan dan Belanja Daerah * (PBI APBD)for members subsidized by local government budgets (or APBD), targeting near-poor populations; (3) Informal non-workers (BP), including employers, investors, pensioners, and veterans; (4) PBPU for informal workers who directly contribute to BPJS; and (5) PPU for formal workers, with contributions shared between employees and employers. Other covariates included the year of data collection, sex, age, urban/rural residence, region, hospital ownership (government or private), and hospital class (A, B, C/D, and specialized).

### Data analysis

The analysis was conducted at the inpatient episode level to retain clinical and financial granularity—such as procedures, severity, length of stay, and costs associated with each hospitalization—and to enhance statistical power in the regression analysis. Multivariate logistic regressions were performed for binary outcome variables, including treatment, severity, and mortality, and reported the results as adjusted odds ratios (AORs). For continuous outcome variables such as length of stay and treatment costs (in the natural log), ordinary least squares (OLS) multivariate regressions were used. For the main results, AORs and OLS coefficients were presented from multivariate regressions. However, results from bivariate regressions or unadjusted odds ratios and coefficients were also provided in Appendix 1 (Supplementary Material). To account for the complex sampling design, robust standard errors were applied using probability weights (using the -pweight- function in Stata), allowing for population-level inferences. All statistical analyses were conducted using Stata 15.1, with a significance level of 5% or lower.

## Results

### Health insurance coverage and hospital claims by membership

**Fig. 1:**
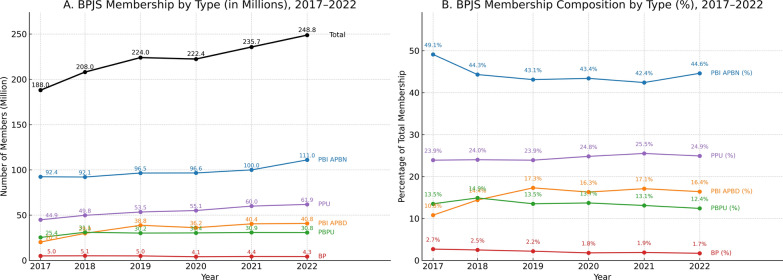
BPJS coverage by membership 2017–2022. Note: PBI APDN = Subsidized members (national budget), PBI APBD = Subsidized members (local government budget), BP = Informal non-worker, PBPU = Informal worker, PPU = Formal worker. Data sources = DJSN Sismonev and BPJS reports

The total number of members steadily increased from 188.0 million in 2017 to 248.8 million in 2022, reflecting a consistent upward trend in coverage (Fig. 1). The proportion of subsidized members funded by the national budget (PBI APBN) remained relatively stable, averaging 44.5%, although there was a slight decline during the study period (Appendix 2 in Supplementary Material). The percentage of subsidized members funded by local government budgets (PBI APBD) was smaller but stable, averaging 15.4%. Informal workers (PBPU) constituted approximately 13.5% of total membership, with minimal fluctuations during this period. Meanwhile, informal non-workers (BP) represented the smallest group, with a consistent average of 2.1%. Notably, formal workers (PPU) showed a gradual increase in coverage with an average of 24.5%.

In terms of the characteristics of hospital claims among BPJS members during 2017–2022, the claim samples include 14,658 cases for IHD and 16,289 cases for stroke (Table 1). Membership patterns reveal that hospital claims were primarily concentrated among informal workers (PBPU) and formal workers (PPU), contributing up to 36.8% of IHD claims and 33.6% of stroke claims. Subsidized members, both under national (PBI APBN) and local government budgets (PBI APBD), have a lower share of claims. Notably, informal non-workers (BP), despite comprising only about 2.1% of BPJS members between 2017 and 2022, accounted for up to 15.7% of stroke claims.

**Table 1:** Characteristics of hospital claim among BPJS members with IHD and stroke in Indonesia 2017–2022

Variables	IHD claim sample		Stroke claim sample	
	n	%	n	%
	[1]	[2]	[3]	[4]
(a) Individual variables				
*Membership*				
PBI APDN	1848	12.6	3100	19.0
PBI APBD	930	6.3	1233	7.6
Informal non-worker	1889	12.9	2553	15.7
Informal worker (PBPU)	5390	36.8	5478	33.6
Formal worker (PPU)	4601	31.4	3925	24.1
*Data year*				
2017	2058	14.0	2435	15.0
2018	2443	16.7	2957	18.2
2019	2678	18.3	2965	18.2
2020	2046	14.0	2431	14.9
2021	2169	14.8	2115	13.0
2022	3264	22.3	3386	20.8
*Sex*				
Female	4776	32.6	7431	45.6
Male	9882	67.4	8858	54.4
*Age group*				
16–49 years	3537	24.1	2906	17.8
50–59 years	5032	34.3	5198	31.9
60 + years	6089	41.5	8185	50.3
*Urbanicity*				
Rural	6536	44.6	9226	56.6
Urban	8122	55.4	7063	43.4
*Region*				
Papua, Maluku, NT	660	4.5	735	4.5
Java, Bali	8251	56.3	9670	59.4
Sumatra	3376	23.0	3259	20.0
Kalimantan	1097	7.5	1245	7.6
Sulawesi	1274	8.7	1380	8.5
*Hospital ownership*				
Government	7904	53.9	9286	57.0
Private	6754	46.1	7003	43.0
*Hospital level*				
Level A	627	4.3	504	3.1
Level B	5989	40.9	6192	38.0
Level C/D	6335	43.2	8781	53.9
Specialized	1707	11.7	812	5.0
(b) Outcome variables				
Treatment (1 = PCI or CT-Scan)	2013	13.7	7422	45.6
Severity (1 = Severe/Moderate)	6701	45.7	7672	47.1
Mortality (1 = Died)	764	5.2	2511	15.4
Length of stay (Mean/SD)	3.78	2.71	5.10	3.96
Cost, Ln (Mean/SD)	15.7	0.9	15.5	0.5
Observations	14,658		16,289	

*IHD* ischemic heart disease, *PBI* Subsidized members, *NT* Nusa Tenggara, *PCI* Percutaneous coronary intervention, *CT Scan* computerized axial tomography of head, *n* sample, *SD* standard deviation

Demographic and regional patterns indicate a significant burden of IHD and stroke among older adults, particularly those aged 60 and above (Table 1). IHD claims were more common among urban residents, while stroke claims were more frequent in rural areas. The majority of claims originated from Java and Bali, Indonesia’s most developed and populous regions. Most cases were treated in government-owned hospitals, with the vast majority of care delivered at Level B and C/D facilities.

In terms of treatment and outcomes (Table 1), 13.7% of IHD patients received PCI, while 45.6% of stroke patients underwent a CT scan. The severity of cases was comparable between the two groups, with about 46% classified as moderate or severe. Mortality was notably higher among stroke patients, with 15.4% of cases resulting in death compared to 5.2% among those with IHD. Stroke patients also had a longer average hospital stay (5.1 days) than IHD patients (3.8 days). The average hospitalization cost, expressed in logarithmic terms, was slightly higher for stroke than for IHD.

### Disparities in health services and outcomes among patients with IHD

Compared to subsidized members (PBI APBN), informal (PBPU) and formal workers (PPU) had higher odds of receiving PCI treatment (AORs: 1.28 and 1.20), although these differences were not statistically significant (Table 2). All three non-subsidized groups—including informal non-workers (BP)—had significantly lower odds of experiencing severe IHD (AORs ranging from 0.66 to 0.76) and lower odds of in-hospital mortality (AORs of 0.61 for BP and 0.64 for PPU). They also had shorter hospital stays—between 0.4 and 0.7 days less on average—but higher odds of incurring greater hospitalization costs, with cost increases ranging from 13 to 24% compared to PBI APBN members.

**Table 2:** Correlates of health services and outcomes among BPJS members with IHD, Indonesia 2017–2022

Variables	PCI		Severity		Died		Length of stay		Cost (Ln)	
	AOR	(SE)	AOR	(SE)	AOR	(SE)	Coef	(SE)	Coef	(SE)
*Membership*										
PBI APDN	Ref		Ref		Ref		Ref		Ref	
PBI APBD	1.22	(0.32)	0.83	(0.12)	0.88	(0.24)	− 0.23	(0.19)	0.02	(0.06)
Informal non-worker	1.32	(0.32)	0.70**	(0.08)	0.61*	(0.12)	− 0.45**	(0.14)	0.23**	(0.04)
Informal worker (PBPU)	1.28	(0.29)	0.76*	(0.08)	0.78	(0.15)	− 0.38**	(0.14)	0.13**	(0.04)
Formal worker (PPU)	1.20	(0.29)	0.66**	(0.08)	0.64*	(0.13)	− 0.65**	(0.14)	0.24**	(0.04)
*Data year*										
2017	Ref		Ref		Ref		Ref		Ref	
2018	1.40	(0.24)	1.08	(0.12)	1.02	(0.22)	− 0.20	(0.12)	0.05	(0.04)
2019	1.58*	(0.29)	0.87	(0.09)	0.92	(0.20)	− 0.41**	(0.12)	0.03	(0.04)
2020	1.64**	(0.30)	0.79*	(0.09)	0.83	(0.25)	− 0.23	(0.14)	0.03	(0.04)
2021	2.13**	(0.45)	1.01	(0.12)	0.89	(0.21)	− 0.12	(0.18)	0.10*	(0.05)
2022	2.05**	(0.35)	0.98	(0.10)	1.26	(0.27)	− 0.41**	(0.13)	0.10**	(0.04)
*Sex*										
Female	Ref		Ref		Ref		Ref		Ref	
Male	2.19**	(0.28)	0.92	(0.06)	1.13	(0.17)	0.06	(0.08)	0.20**	(0.02)
*Age group*										
16–49 years	Ref		Ref		Ref		Ref		Ref	
50–59 years	0.94	(0.14)	1.45**	(0.14)	1.55*	(0.34)	0.09	(0.11)	0.07	(0.04)
60 + years	0.54**	(0.09)	1.96**	(0.19)	3.69**	(0.78)	0.40**	(0.12)	0.05	(0.04)
Urbanicity										
Rural	Ref		Ref		Ref		Ref		Ref	
Urban	2.29**	(0.38)	1.03	(0.08)	0.77	(0.11)	− 0.19*	(0.09)	0.22**	(0.03)
*Region*										
Papua, Maluku, NT	Ref		Ref		Ref		Ref		Ref	
Java, Bali	2.11*	(0.71)	1.43*	(0.22)	0.70	(0.23)	− 0.14	(0.23)	0.07	(0.04)
Sumatra	2.21*	(0.77)	0.79	(0.13)	0.42*	(0.15)	0.11	(0.24)	− 0.04	(0.05)
Kalimantan	1.47	(0.56)	1.58*	(0.29)	0.45*	(0.18)	− 0.33	(0.25)	0.04	(0.05)
Sulawesi	1.21	(0.46)	1.33	(0.23)	0.80	(0.31)	0.46	(0.25)	− 0.05	(0.05)
*Hospital ownership*										
Government	Ref		Ref		Ref		Ref		Ref	
Private	2.52**	(0.33)	1.20*	(0.09)	0.83	(0.13)	− 0.62**	(0.10)	0.16**	(0.03)
*Hospital level*										
Level A	Ref		Ref		Ref		Ref		Ref	
Level B	0.34**	(0.05)	0.61**	(0.07)	1.14	(0.28)	− 0.39*	(0.18)	− 0.81**	(0.05)
Level C/D	0.02**	(0.01)	0.55**	(0.07)	1.13	(0.32)	− 0.43*	(0.20)	− 1.48**	(0.06)
Specialized	0.36**	(0.09)	0.65	(0.15)	0.46	(0.25)	− 1.11**	(0.26)	− 0.40**	(0.11)
Constant	0.05**	(0.02)	0.95	(0.23)	0.06**	(0.03)	4.98**	(0.34)	16.11**	(0.09)
Observations	14,658		14,658		14,658		14,658		14,658	

*PCI* percutaneous coronary intervention, *AOR* adjusted odds ratio, *Coef* coefficient, *SE* standard errors, *Ref* reference, *NT* Nusa Tenggara, *PBI* subsidized members, *Ln* natural log. Columns 1–3 used logit regressions, columns 4–5 used OLS regressions; pooled analyses in Stata 15. Robust SE in parentheses. **p < 0.01, *p < 0.05

Several demographic and geographic characteristics were associated with variation in IHD outcomes. Males had significantly higher odds of receiving PCI compared to females (AOR: 2.19), and urban residents had higher odds than rural residents (AOR: 2.29). Older individuals, particularly those aged 60 and above, had substantially higher odds of severe IHD (AOR: 1.96) and mortality (AOR: 3.69) compared to those aged 16–49. Regional differences were also observed, with patients from Java-Bali and Sumatra showing higher odds of severe IHD relative to those from Papua, Maluku, and Nusa Tenggara.

Hospital ownership and class also influenced patient outcomes. Patients in private hospitals had higher odds of receiving PCI (AOR: 2.52) and experienced shorter lengths of stay (by 0.62 days on average) compared to those in government hospitals. Patients treated in Level A hospitals had lower odds of receiving PCI (AOR: 0.34) and shorter stays, while those in Level C/D hospitals had even shorter stays and significantly lower odds of incurring high costs (cost coefficient: − 1.48).

### Disparities in health services and outcomes among patients with stroke

Compared to PBI APBN members, the odds of receiving a CT scan did not differ significantly across other membership types, with AORs close to 1 (Table 3). However, informal non-workers (BP) had significantly lower odds of in-hospital mortality (AOR: 0.66), and both informal (PBPU) and formal workers (PPU) also showed lower odds of death (AORs: 0.73 and 0.54, respectively), suggesting better survival among non-subsidized groups. In terms of hospital stay and costs, BP members had longer stays and higher odds of incurring greater hospitalization costs, while PBPU and PPU members also had higher costs but mixed results for length of stay.

**Table 3:** Correlates of health services and outcomes among BPJS members with stroke, Indonesia 2017–2022

Variables	CT scan		Severity		Died		Length of stay		Cost (Ln)	
	AOR	(SE)	AOR	(SE)	AOR	(SE)	Coef	(SE)	Coef	(SE)
*Membership*										
PBI APDN	Ref		Ref		Ref		Ref		Ref	
PBI APBD	0.90	(0.13)	1.10	(0.16)	1.02	(0.19)	− 0.00	(0.22)	0.08*	(0.04)
Informal non-worker	1.05	(0.11)	1.01	(0.10)	0.66**	(0.09)	0.71**	(0.18)	0.30**	(0.02)
Informal worker (PBPU)	0.98	(0.09)	0.94	(0.08)	0.73**	(0.08)	0.45**	(0.13)	0.14**	(0.02)
Formal worker (PPU)	0.99	(0.09)	0.95	(0.09)	0.54**	(0.07)	0.28	(0.15)	0.31**	(0.02)
*Data year*										
2017	Ref		Ref		Ref		Ref		Ref	
2018	1.27*	(0.13)	0.98	(0.10)	0.95	(0.14)	− 0.33	(0.18)	− 0.01	(0.02)
2019	1.44**	(0.15)	0.97	(0.10)	1.25	(0.17)	− 0.32	(0.20)	0.01	(0.02)
2020	1.82**	(0.22)	1.02	(0.12)	1.14	(0.19)	− 0.59**	(0.22)	− 0.03	(0.02)
2021	2.19**	(0.25)	1.13	(0.13)	1.01	(0.15)	− 0.37	(0.20)	0.04	(0.02)
2022	3.35**	(0.34)	1.14	(0.12)	0.87	(0.12)	− 0.60**	(0.17)	0.07**	(0.02)
*Sex*										
Female	Ref		Ref		Ref		Ref		Ref	
Male	1.12	(0.07)	0.94	(0.06)	1.19	(0.11)	− 0.18	(0.11)	− 0.00	(0.01)
*Age group*										
16–49 years	Ref		Ref		Ref		Ref		Ref	
50–59 years	1.00	(0.10)	0.99	(0.10)	0.86	(0.13)	0.09	(0.18)	− 0.01	(0.03)
60 + years	0.88	(0.09)	0.99	(0.10)	1.29	(0.19)	0.15	(0.17)	0.02	(0.03)
Urbanicity										
Rural	Ref		Ref		Ref		Ref		Ref	
Urban	1.62**	(0.12)	1.20*	(0.09)	0.86	(0.09)	0.14	(0.12)	0.10**	(0.02)
*Region*										
Papua, Maluku, NT										
Java, Bali	1.93**	(0.29)	0.80	(0.13)	1.09	(0.26)	− 0.78**	(0.28)	− 0.04	(0.03)
Sumatra	1.74**	(0.27)	0.50**	(0.08)	1.10	(0.26)	− 0.58	(0.30)	− 0.11**	(0.03)
Kalimantan	1.58*	(0.34)	0.78	(0.17)	1.02	(0.28)	− 0.47	(0.36)	− 0.08	(0.04)
Sulawesi	1.40*	(0.24)	0.68*	(0.13)	1.12	(0.29)	0.07	(0.34)	− 0.07	(0.04)
*Hospital ownership*										
Government	Ref		Ref		Ref		Ref		Ref	
Private	0.85*	(0.06)	1.33**	(0.10)	0.78*	(0.08)	− 0.31*	(0.13)	0.07**	(0.02)
*Hospital level*										
Level A	Ref		Ref		Ref		Ref		Ref	
Level B	0.96	(0.15)	0.52**	(0.08)	0.65**	(0.11)	− 2.29**	(0.35)	− 0.57**	(0.05)
Level C/D	0.59**	(0.10)	0.32**	(0.05)	0.49**	(0.09)	− 3.20**	(0.35)	− 0.98**	(0.05)
Specialized	2.60**	(0.62)	1.75*	(0.42)	0.35**	(0.10)	− 1.24*	(0.49)	− 0.35**	(0.06)
Constant	0.31**	(0.08)	2.52**	(0.65)	0.39**	(0.13)	8.47**	(0.49)	16.07**	(0.06)
Observations	16,289		16,289		16,289		16,289		16,288	

*CT Scan* computerized axial tomography of head, *AOR* adjusted odds ratio, *Coef* coefficient, *SE* standard errors, *Ref* reference, *NT* Nusa Tenggara, *PBI* subsidized members, *Ln* Natural log. Columns 1–3 used logit regressions, columns 4–5 used OLS regressions; pooled analyses in Stata 15. Robust SE in parentheses. **p < 0.01, *p < 0.05

Stroke-related outcomes were also associated with key demographic and geographic variables (Table 3). Although sex and age group differences in outcomes were observed, none reached statistical significance. Urban residents had higher odds of receiving CT scans (AOR: 1.62) compared to rural residents, but also higher odds of severe stroke and higher hospitalization costs. Regionally, patients from Java-Bali, Sumatra, Sulawesi, and Kalimantan had higher odds of receiving CT scans (AORs: 1.58–1.93), while stroke severity was significantly lower among those from Sumatra (AOR: 0.50) and Sulawesi (AOR: 0.68) compared to patients from eastern Indonesia.

Hospital ownership and level also influenced stroke care and outcomes. Patients in private hospitals had lower odds of receiving CT scans (AOR: 0.85) but higher odds of experiencing severe stroke (AOR: 1.33) compared to those in government hospitals (Table 3). Private facilities were also associated with shorter hospital stays and slightly higher hospitalization costs. Patients treated in specialized hospitals had higher odds of receiving CT scans (AOR: 2.60) and severe stroke (AOR: 1.75), while those in lower-level hospitals (Level B and C/D) had lower odds of both severe stroke (AORs: 0.52 and 0.32) and high costs. These lower-tier hospitals also showed significantly shorter lengths of stay and lower costs, possibly reflecting differences in care intensity and available services.

## Discussion

The disparities in health service utilization among BPJS members in Indonesia are starkly highlighted by the differences in claim proportions across membership types. Despite representing nearly 50% of the total BPJS membership, the PBI APBN group (i.e. the poorest members) accounted for only 12% of IHD claims and 19% of stroke claims. In contrast, the informal non-worker (BP) group, which constitutes just 2% of the membership, was responsible for 12.9% of IHD claims and 15.7% of stroke claims. Similarly, the informal worker (PBPU) group, making up 13.5% of total members, accounted for 36.8% of IHD claims and 33.6% of stroke claims. These patterns suggest that socioeconomic status significantly influences healthcare utilization, with non-subsidized members accessing more advanced treatments and services. This aligns with findings from other studies indicating that higher socioeconomic groups often have better access to healthcare services due to fewer financial barriers and greater awareness of healthcare needs [6, 15]. Addressing these disparities is crucial for achieving equitable healthcare access and improving outcomes for all BPJS members.

The findings reveal substantial disparities in access to health services and outcomes for IHD and stroke between different BPJS membership types, highlighting socioeconomic inequities in the Indonesian healthcare system. PBI APBN members, representing the poorest segment of the population who received subsidies through the national budget, consistently showed less favorable outcomes compared to other membership types [11]. For IHD, PBI APBN members had lower access to advanced procedures like PCI and were more likely to experience severe cases and higher mortality rates [6]. In contrast, informal non-workers (BP) and formal workers (PPU), who tend to have higher socioeconomic status and contribute directly to BPJS, had significantly lower odds of severe IHD cases and mortality, despite similar levels of access to PCI treatment. Additionally, hospital length of stay (LOS) is significantly longer for PBI APBN members with IHD compared to other groups, possibly reflecting delays in receiving care or less efficient hospital processes for poorer patients. In contrast, BP, PBPU, and PPU members experience shorter hospital stays, which may be associated with more efficient care pathways and the ability to access higher-quality facilities [11].

For stroke patients, a different pattern emerges: PBI APBN members had significantly shorter hospital stays compared to their wealthier counterparts. This could suggest a range of factors, such as earlier discharge due to resource constraints or the inability to access extended care that might be more available to wealthier groups [22]. Despite similar access to essential diagnostic tools like CT scans, PBI APBN members have poorer survival outcomes, which may point to differences in the quality or continuity of care received [22]. Regarding hospital claim costs, which represent the payments BPJS makes to providers rather than out-of-pocket expenses for patients, informal non-workers (BP), informal workers (PBPU), and formal workers (PPU) incurred significantly higher costs compared to PBI APBN members. This disparity could be attributed to higher unit costs associated with the treatment of non-subsidized members, who are more often treated in Class 1 or Class 2 hospitals, which are generally more expensive. In contrast, many PBI APBN and PBI APBD members are treated in Class 3 hospitals, which have lower service costs [23]. Thus, while the BPJS system covers a significant portion of care for all membership types, the variation in claim costs reflects differences in the type and intensity of services provided to wealthier members, underscoring ongoing challenges in achieving equitable resource distribution across socioeconomic groups.

From a policy perspective, the findings offer valuable insights for Indonesia and other LMICs) working toward achieving UHC. With the high and increasing burden of CVDs, such as IHD and stroke, achieving equitable access to healthcare has become more critical than ever. The findings suggest that PBI APBN members, the poorest segment of BPJS beneficiaries, have lower access to advanced treatments like PCI and experience poorer outcomes, especially for IHD. Policymakers should prioritize targeted interventions to support this group, such as increasing subsidies, ensuring better access to higher-level hospitals, and improving the quality of care in Class 3 facilities where many PBI APBN members receive treatment [17, 23]. Additionally, efforts to streamline care processes for these members could reduce prolonged hospital stays and improve efficiency in care delivery. Given that higher unit costs for wealthier members are often due to treatment in Class 1 or Class 2 hospitals, policies aimed at redistributing resources to improve services in Class 3 hospitals could help address the disparities in healthcare costs and outcomes. Fortunately, from mid 2024, the new initiative of Standard Inpatient Class (*Kelas Rawat Inap Standard* [KRIS]) by the Ministry of Health aims to ensure equal treatment for all patients [24]. By focusing on these areas, Indonesia can move closer to achieving equitable health coverage and improving outcomes for all its citizens, regardless of socioeconomic status [25–27].

The study has several limitations that should be considered when interpreting the findings. First, the cross-sectional design limits the ability to infer causal relationships between BPJS membership types and health outcomes. The study assessed a limited set of predefined outcomes. As an exploratory analysis, no formal correction for multiple testing was applied. Therefore, findings should be interpreted with caution, particularly where *p* values approach the significance threshold. Second, while the study used a random sample of BPJS claim data (about 1% of total claims), it may not fully capture all relevant factors influencing healthcare access and outcomes, such as patient behaviors, variations in the quality of care, or socio-cultural barriers to seeking care [28, 29]. Additionally, the use of administrative claim data, while comprehensive, is subject to potential inaccuracies in coding and reporting, which could affect the precision of the identified associations. Third, some of the claim data were collected during the COVID-19 pandemic, which may have influenced healthcare utilization and outcomes due to service disruptions or changes in clinical protocols [30, 31]. Finally, although the study adjusted for hospital ownership and class, it did not explore other potentially important factors like regional healthcare infrastructure and provider availability, which could also contribute to the observed disparities [32]. Addressing these limitations in future research could offer a more detailed understanding of the mechanisms driving healthcare inequities among BPJS members.

## Conclusions

This study highlights persistent disparities in health services and outcomes for IHD and stroke across BPJS membership types in Indonesia. For IHD, members under the national subsidy scheme (PBI APBN) had lower odds of receiving PCI compared to non-subsidized groups, although the difference was not statistically significant. In contrast, access to CT scans among stroke patients did not vary notably by membership type. Hospital length of stay differed by condition and membership: PBI APBN members tended to stay longer for IHD and shorter for stroke. Non-subsidized members—including informal non-workers (BP), informal workers (PBPU), and formal workers (PPU)—incurred significantly higher hospitalization costs for both conditions. In terms of outcomes, non-subsidized IHD patients had lower odds of severe illness and in-hospital mortality, with particularly reduced mortality observed among BP and PPU members. Among stroke patients, while severity was similar across groups, all three non-subsidized categories had significantly lower odds of death compared to PBI APBN members. These findings underscore socioeconomic disparities within the national health insurance system and point to a need for targeted improvements in access, care efficiency, and quality—especially for the poorest segments. Simultaneously, broader efforts to prevent IHD and stroke through community-based healthy lifestyle promotion (e.g. E(e)SEEDi) and stronger tobacco control policies may help reduce these disparities over time.

## Supplementary Information

Additional file 1.

## Data Availability

Available from the authors upon reasonable request.

## Abbreviations

AOR: Adjusted odds ratio
BP: Informal non-worker (*Bukan Pekerja*)
BPJS: National Health Insurance Agency (*Badan Penyelenggara Jaminan Sosial*)
CVD: Cardiovascular diseases
CT: Computed tomography
RG: Diagnosis-related group
ICD-10: International classification of diseases, 10th revision
IHD: Ischemic heart disease
INA-CBGs: Indonesia case-based groups (Indonesia’s DRG-based hospital payment system)
LOS: Length of stay
NT: Nusa Tenggara
OLS: Ordinary least squares
PBPU: *Pekerja Bukan Penerima Upah* (Informal worker)
PCI: Percutaneous coronary intervention
PBI APBN: *Penerima Bantuan Iuran Anggaran Pendapatan dan Belanja Negara*
PBI APBD: *Penerima Bantuan Iuran Anggaran Pendapatan dan Belanja Daerah*
PPU: *Pekerja Penerima Upah* (Formal worker)
UHC: Universal health coverage
