# Supplementary material for: Disparities in health services and outcomes by National Health Insurance membership type for ischemic heart disease and stroke in Indonesia: analysis of claims, 2017–2022
Source: Glob Health Res Policy. 2025 Aug 1;10:33. doi: 10.1186/s41256-025-00432-y (PMC12315411; doi:10.1186/s41256-025-00432-y)
Supplement: Supplementary file 1 — Additional file 1. [file 41256_2025_432_MOESM1_ESM.pdf]

Disparities in Health Services and Outcomes by National Health Insurance Membership Type for Ischemic Heart Disease and Stroke in Indonesia: Analysis of Claims, 2017–2022
Darmawan et al. (2025)

Appendix 1: Unadjusted regressions

(a) IHD

	PCI		Severity		Died		Length of stay		Cost (Ln)	
Variables	AOR	(SE)	AOR	(SE)	AOR	(SE)	Coef	(SE)	Coef	(SE)
Membership										
PBI APDN	Ref		Ref		Ref		Ref		Ref	
PBI APBD	2.81**	(0.77)	0.81	(0.11)	0.63	(0.17)	-0.35	(0.19)	0.28**	(0.07)
Informal non-worker	1.77*	(0.45)	0.96	(0.11)	0.80	(0.16)	-0.32*	(0.15)	0.45**	(0.05)
Informal worker (PBPU)	2.32**	(0.55)	0.90	(0.09)	0.69	(0.13)	-0.47**	(0.14)	0.39**	(0.05)
Formal worker (PPU)	2.61**	(0.62)	0.64**	(0.06)	0.41**	(0.08)	-0.81**	(0.13)	0.50**	(0.05)
Constant	0.08**	(0.02)	1.04	(0.09)	0.09**	(0.01)	4.18**	(0.11)	15.40**	(0.04)
Observations	14,658		14,658		14,658		14,658		14,658	

(b) Stroke

	CT scan		Severity		Died		Length of stay		Cost (Ln)	
Variables	AOR	(SE)	AOR	(SE)	AOR	(SE)	Coef	(SE)	Coef	(SE)
Membership										
PBI APDN	Ref		Ref		Ref		Ref		Ref	
PBI APBD	1.13	(0.17)	1.19	(0.18)	1.00	(0.19)	0.26	(0.23)	0.15**	(0.04)
Informal non-worker	1.11	(0.11)	1.15	(0.11)	0.74*	(0.09)	1.01**	(0.19)	0.40**	(0.02)
Informal worker (PBPU)	1.01	(0.09)	1.01	(0.09)	0.71**	(0.08)	0.50**	(0.14)	0.18**	(0.02)
Formal worker (PPU)	1.14	(0.10)	1.06	(0.10)	0.49**	(0.06)	0.40*	(0.16)	0.37**	(0.02)
Constant	0.81**	(0.06)	0.91	(0.06)	0.26**	(0.02)	4.66**	(0.10)	15.34**	(0.01)
Observations	16,289		16,289		16,289		16,289		16,288	

Note: PCI= Percutaneous coronary intervention, CT Scan=Computerized axial tomography of head, AOR=Adjusted Odds Ratio, Coef=Coefficient, SE=Standard errors, Ref=Reference, PBI= Subsidized members, Ln=Natural log. Columns 1-3 used logit regressions, columns 4-5 used OLS regressions; pooled analyses in Stata 15. Robust SE in parentheses. ** p<0.01, * p<0.05

Appendix 2: BPJS coverage by membership 2017-2022

Year	Total (million)	PBI APBN (million)		PBI APBD (million)		Informal workers (PBPU) (million)		Informal Non workers (BP) (million)		Formal workers (PPU) (million)	
	N	n	%	n	%	n	%	n	%	n	%
2017	188.0	92.4	49.1%	20.3	10.8%	25.4	13.5%	5.0	2.7%	44.9	23.9%
2018	208.1	92.1	44.3%	29.9	14.4%	31.1	14.9%	5.1	2.5%	49.8	24.0%
2019	224.1	96.5	43.1%	38.8	17.3%	30.2	13.5%	5.0	2.2%	53.5	23.9%
2020	222.5	96.6	43.4%	36.2	16.3%	30.4	13.7%	4.1	1.8%	55.1	24.8%
2021	235.7	100.0	42.4%	40.4	17.1%	30.9	13.1%	4.4	1.9%	60.0	25.5%
2022	248.8	111.0	44.6%	40.8	16.4%	30.8	12.4%	4.3	1.7%	61.9	24.9%
Average			44.5%		15.4%		13.5%		2.1%		24.5%

Sources: DJSN Sismonev and BPJS reports
